# Supplementary material for: Differential Genetic Architecture of Insulin Resistance (HOMA-IR) Based on Obesity Status: Evidence from a Large-Scale GWAS of Koreans
Source: Curr Issues Mol Biol. 2025 Jun 16;47(6):461. doi: 10.3390/cimb47060461 (PMC12191708; doi:10.3390/cimb47060461)
Supplement: Supplementary file 1 [file cimb-47-00461-s001.zip › Supplementary Figure.pdf]

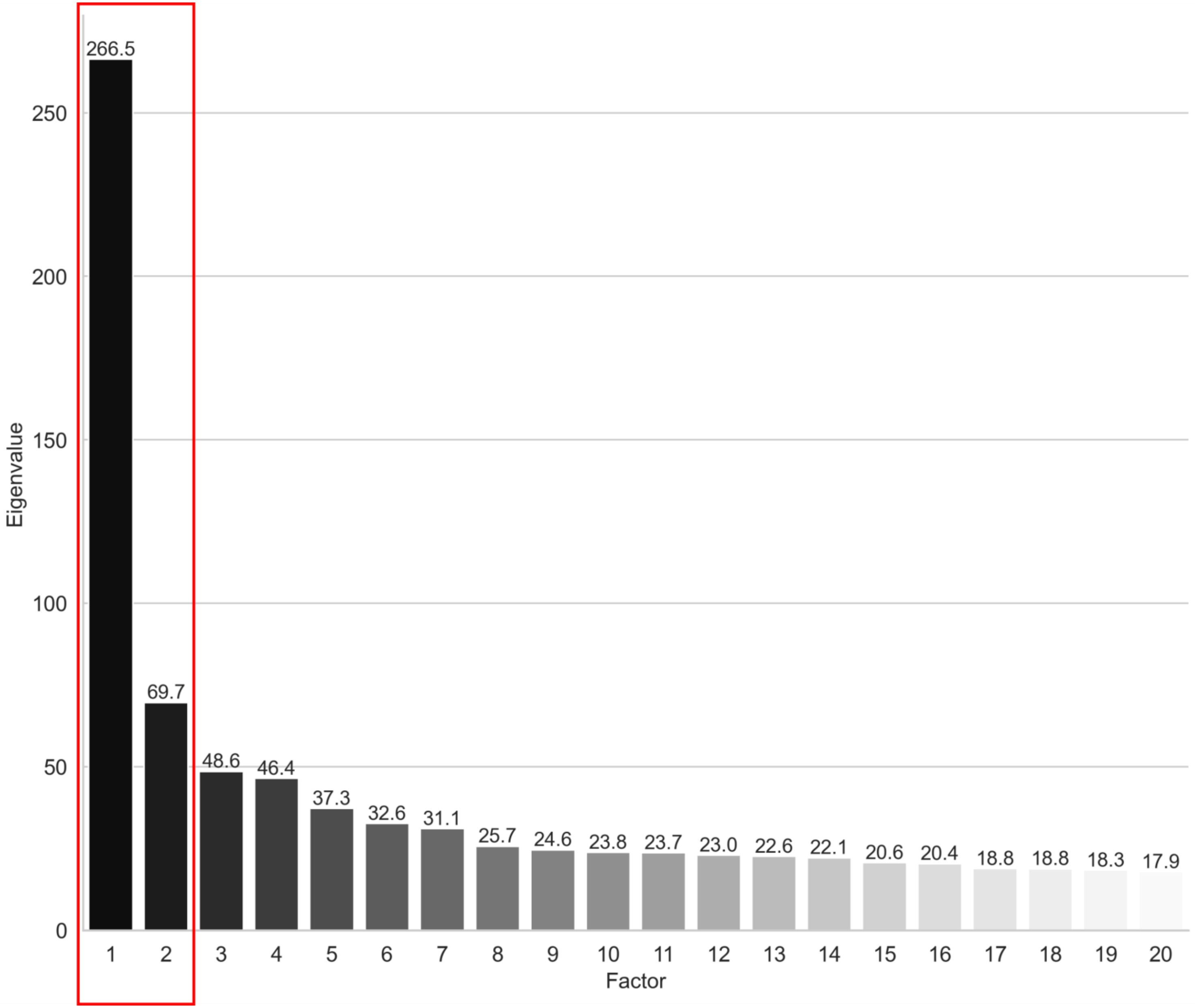

Supplementary Figure S1. Eigenvalues of the top 20 principal components derived from the KoGES cohort.

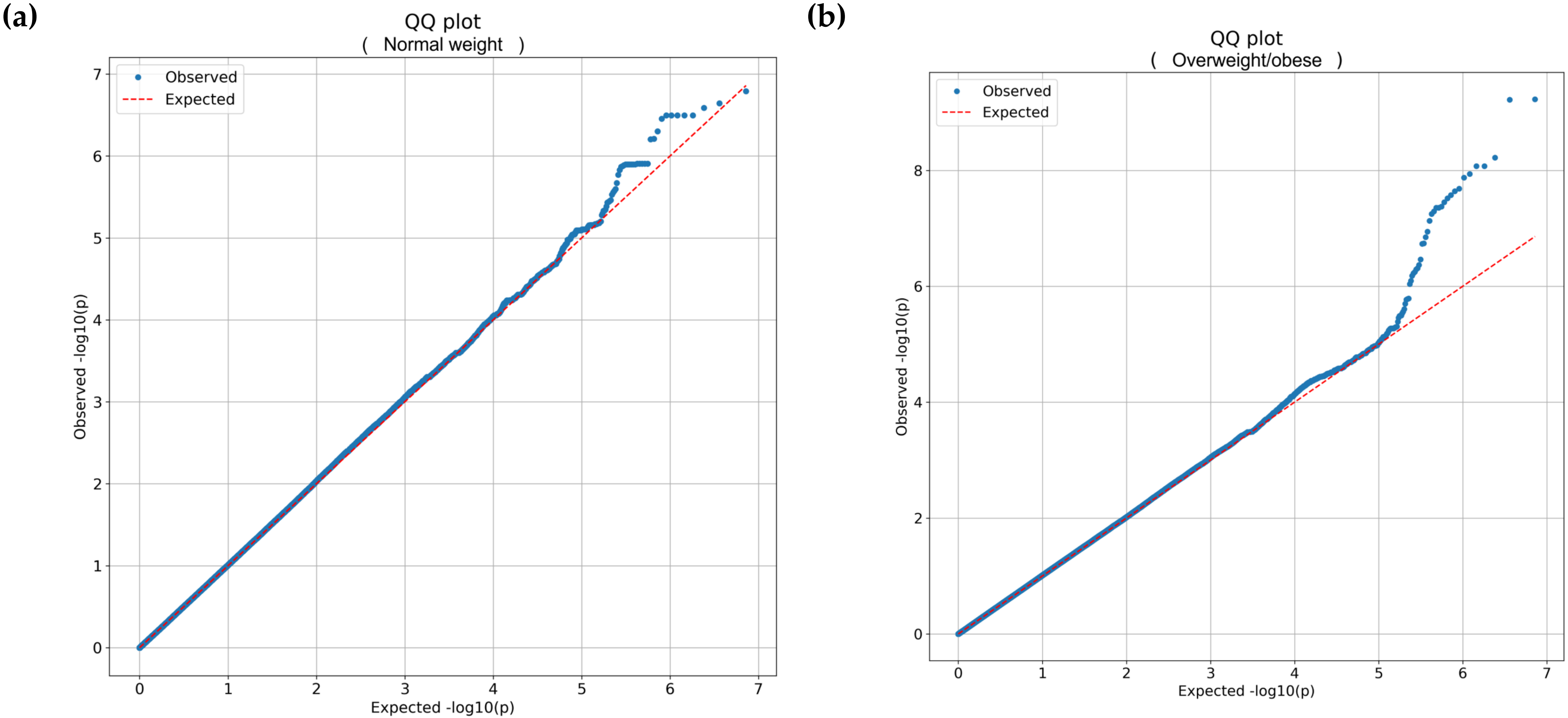

Supplementary Figure S2. QQ plots. Observed $-\log_{10}$(p-values) are plotted against expected $-\log_{10}$(p-values) under the null hypothesis. The diagonal line represents the null distribution. The genomic inflation factor (λGC) indicates the extent of population stratification or systematic bias. (a) Normal weight group (λGC = 1.005); (b) Overweight/obese group (λGC = 1.022).
