# Supplementary figures and images for: Differential Genetic Architecture of Insulin Resistance (HOMA-IR) Based on Obesity Status: Evidence from a Large-Scale GWAS of Koreans
Source: Curr Issues Mol Biol. 2025 Jun 16;47(6):461. doi: 10.3390/cimb47060461 (PMC12191708; doi:10.3390/cimb47060461)

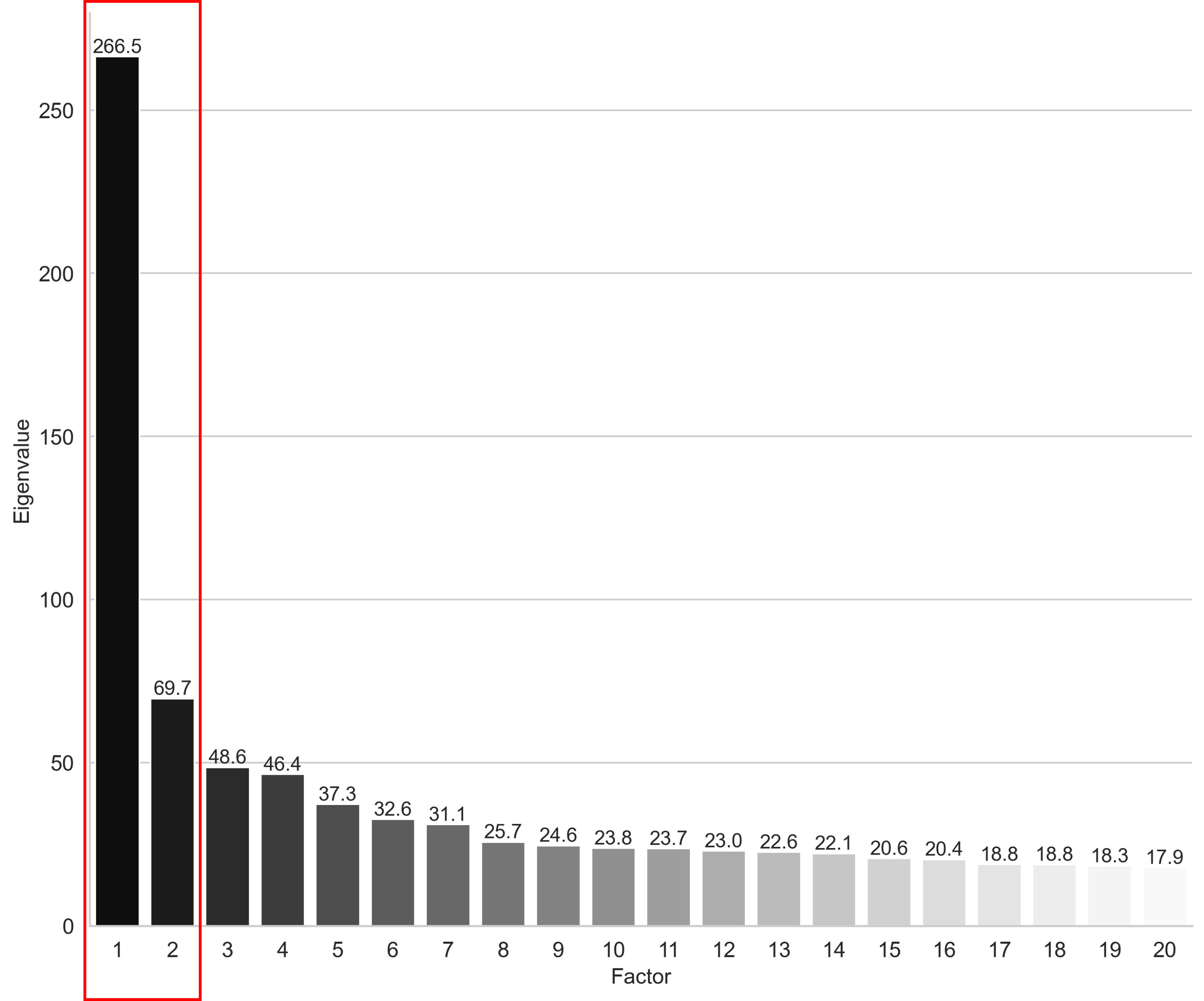

Supplement: Supplementary file 1 [file cimb-47-00461-s001.zip › Figure S1.png]

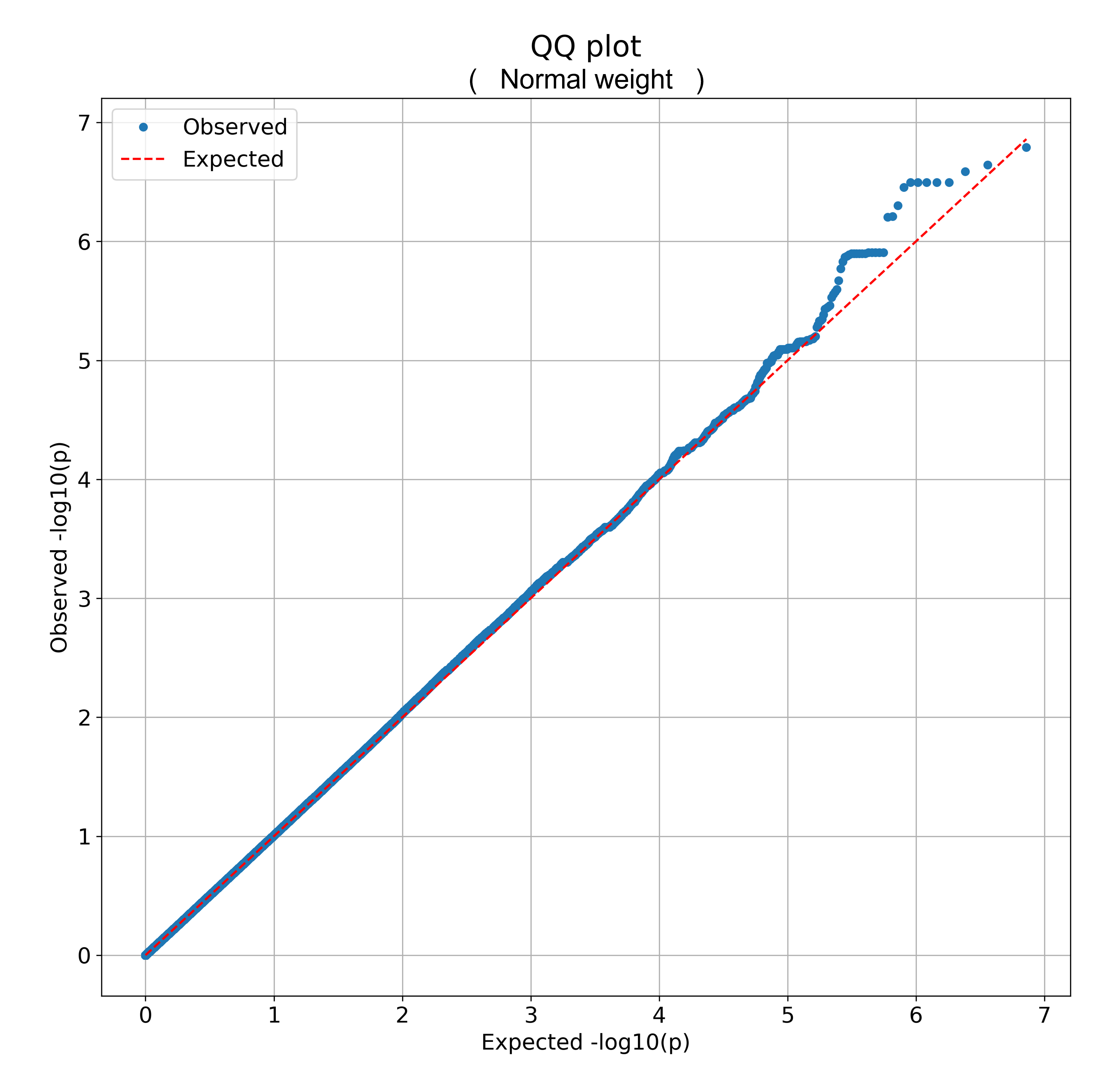

Supplement: Supplementary file 1 [file cimb-47-00461-s001.zip › Figure S2a.png]

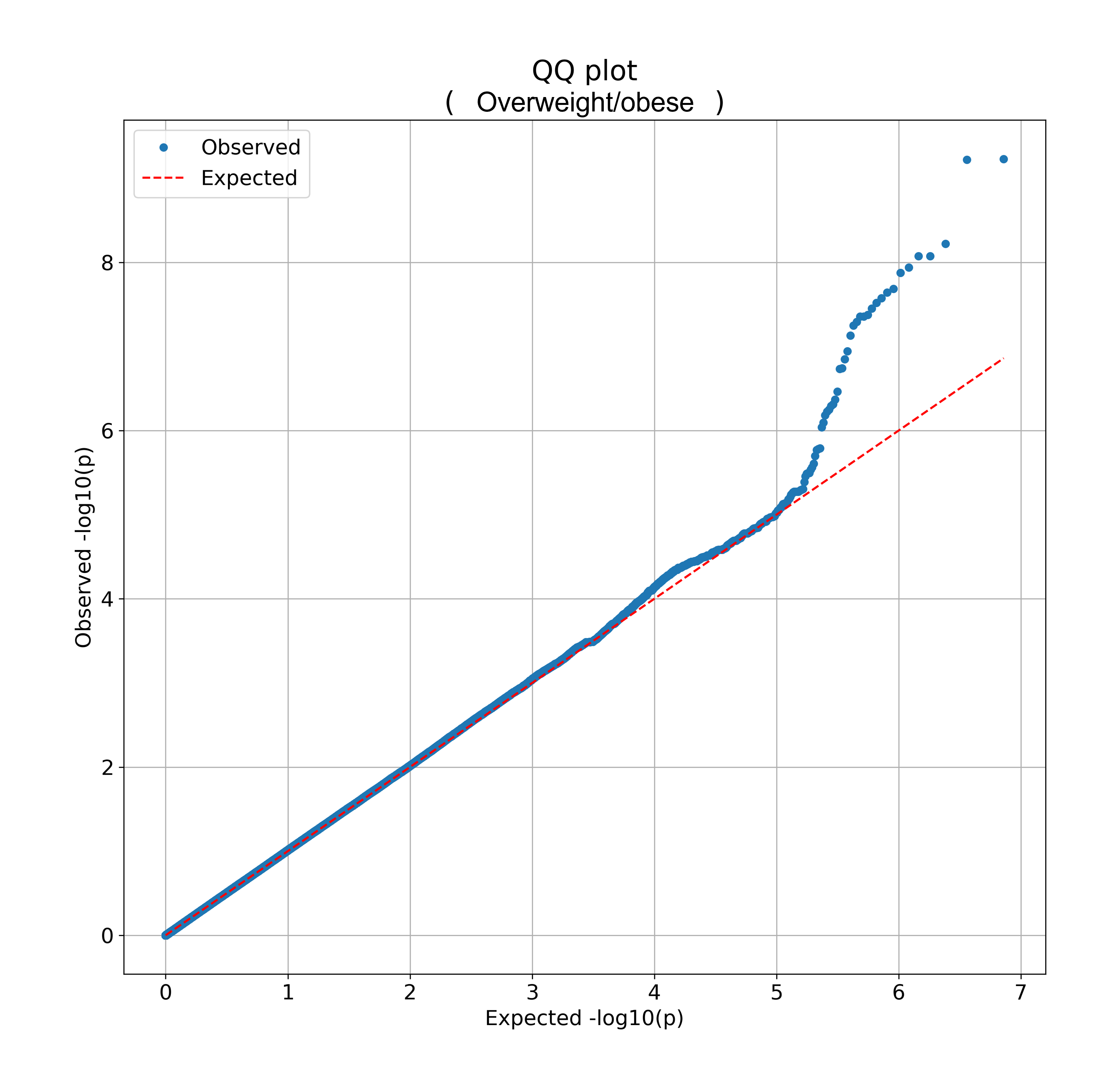

Supplement: Supplementary file 1 [file cimb-47-00461-s001.zip › Figure S2b.png]
